# Torpor induces reversible tau hyperphosphorylation and accumulation in mice expressing human tau

**DOI:** 10.1186/s40478-024-01800-4

**Published:** 2024-06-04

**Authors:** C. F. de Veij Mestdagh, M. E. Witte, W. Scheper, A. B. Smit, R. H. Henning, R. E. van Kesteren

**Affiliations:** 1 Department of Molecular and Cellular Neurobiology, Center for Neurogenomics and Cognitive Research, Vrije Universiteit Amsterdam, Amsterdam, The Netherlands; 2 Department of Clinical Pharmacy and Pharmacology, University Medical Center Groningen, Groningen, The Netherlands; 3 Alzheimer Center Amsterdam, Amsterdam UMC location VUmc, Amsterdam, The Netherlands; 4 Department of Molecular Cell Biology and Immunology, MS Center, Amsterdam UMC location VUmc, Amsterdam, The Netherlands; 5 Department of Human Genetics, Amsterdam UMC - location Vrije Universiteit, Amsterdam, The Netherlands; 6 Department of Functional Genomics, Center for Neurogenomics and Cognitive Research, Vrije Universiteit Amsterdam, Amsterdam, The Netherlands

**Keywords:** Mouse torpor, Hibernation, Alzheimer’s Disease, Human tau expressing mice, Tau hyperphosphorylation, AT8 somato-dendritic accumulation

## Abstract

Tau protein hyperphosphorylation and aggregation are key pathological events in neurodegenerative tauopathies such as Alzheimer’s disease. Interestingly, seasonal hibernators show extensive tau hyperphosphorylation during torpor, i.e., the hypothermic and hypometabolic state of hibernation, which is completely reversed during arousal. Torpor-associated mechanisms that reverse tau hyperphosphorylation may be of therapeutic relevance, however, it is currently not known to what extent they apply to human tau. Here we addressed this issue using daily torpor in wildtype mice that express mouse tau (mtau) and in mice that lack mtau expression and instead express human tau (htau). AT8, AT100 and Ser396 immunoblotting and immunohistochemistry were used to assess tau (hyper)phosphorylation at clinically relevant phosphorylation sites. We found that torpor robustly and reversibly increases the levels of phosphorylated tau in both mtau and htau mice. Immunohistochemistry revealed four brain areas that show prominent tau phosphorylation: the hippocampus, posterior parietal cortex, piriform cortex and cortical amygdala. Whereas wildtype mice primarily showed increased levels of diffusely organized hyperphosphorylated tau during torpor, htau mice contained clear somato-dendritic accumulations of AT8 reactivity resembling tau pre-tangles as observed in the Alzheimer brain. Interestingly, AT8-positive accumulations disappeared upon arousal, and tau phosphorylation levels at 24 h after arousal were lower than observed at baseline, suggesting a beneficial effect of torpor-arousal cycles on preexisting hyperphosphorylated tau. In conclusion, daily torpor in mice offers a quick and standardized method to study tau phosphorylation, accumulation and clearance in mouse models relevant for neurodegeneration, as well as opportunities to discover new targets for the treatment of human tauopathies.

**Supplementary Information:**

The online version contains supplementary material available at 10.1186/s40478-024-01800-4.

## Introduction

Tau is the collection of proteins generated from the MAPT gene. Tau hyperphosphorylation and subsequent aggregate formation are closely linked to severe neurodegenerative tauopathies such as Alzheimer’s disease (AD) [1]. In AD, tau becomes hyperphosphorylated, by which it dissociates from microtubules and accumulates in the cytoplasm where it can form paired helical filaments, AT8 detectable pre-tangles and eventually neurofibrillary tangles [2]. This results in loss-of-function phenotypes as tau no longer performs microtubule stabilization, as well as gain-of-function phenotypes resulting from toxicity of tau aggregates [3]. Together, these conditions may lead to functional impairment of neurons and neurodegeneration [4].

In the last decades, research in seasonal hibernators such as the arctic ground squirrel, Syrian hamster and black bear has demonstrated that during torpor, i.e., long-term periods of hypothermia and -metabolism, hibernators undergo tau hyperphosphorylation and conformational changes similar to those seen in AD [5–9]. Remarkably, tau hyperphosphorylation is completely reversed during arousal, i.e., the periodic rapid rewarming and reactivation of mitochondria in between torpor bouts, without any signs of post-hibernation damage. It has been suggested that hibernation employs specific mechanisms to reverse tau hyperphosphorylation, which might hold promise for the treatment of tauopathies in humans. Obtaining such insights from seasonal hibernators is however very challenging, mainly because they are non-standard laboratory animals that lack proper genomic and proteomic annotation and because of lack of (genetic) disease models. The notion that laboratory mice are capable of daily torpor [10–12], showing bouts of torpor and arousal of several hours, offers the unique opportunity to explore torpor-associated tau hyperphosphorylation in a standardized manner, with numerous disease models available.

In this study we determined whether daily torpor in mice induces tau hyperphosphorylation in the brain of wildtype mice expressing mouse tau (mtau). Furthermore, we took advantage of a transgenic mouse line expressing human tau (htau) in the absence of mtau to uniquely assess the effects of torpor on human tau. We measured the onset, level and reversibility of tau phosphorylation using immunoblotting and immunostaining with AT8, AT100 and Ser396, antibodies that detect phosphorylation at well-documented abnormal tau phosphorylation sites (AT8: Ser202 and Thr205, AT100: Thr212 and Ser214) and are commonly used for post-mortem biochemical staging of AD [13]. We show that torpor induces robust and reversible tau hyperphosphorylation in both mtau and htau mice. Relatively strong tau phosphorylation was observed in the hippocampus, posterior parietal cortex, piriform cortex and cortical amygdala. In contrast to the diffuse distribution of hyperphosphorylated tau in mtau mice, htau mice displayed somato-dendritic accumulations of AT8-detectable tau in all four brain areas, resembling the appearance of tau pre-tangles in AD brains [14, 15]. Like total AT8 reactivity, these accumulations disappeared 24 h after torpor. Interestingly, tau phosphorylation levels were lower 24 h after torpor than in euthermic controls. Together these data show that torpor in mice is a robust and rapid method to induce and study tau hyperphosphorylation and accumulation, offering promising opportunities to discover novel tau clearance mechanisms that may be relevant for the treatment of human neurodegenerative disease.

## Results

### Torpor induces reversible tau hyperphosphorylation in the hippocampus of mtau and htau mice

**Fig. 1:**
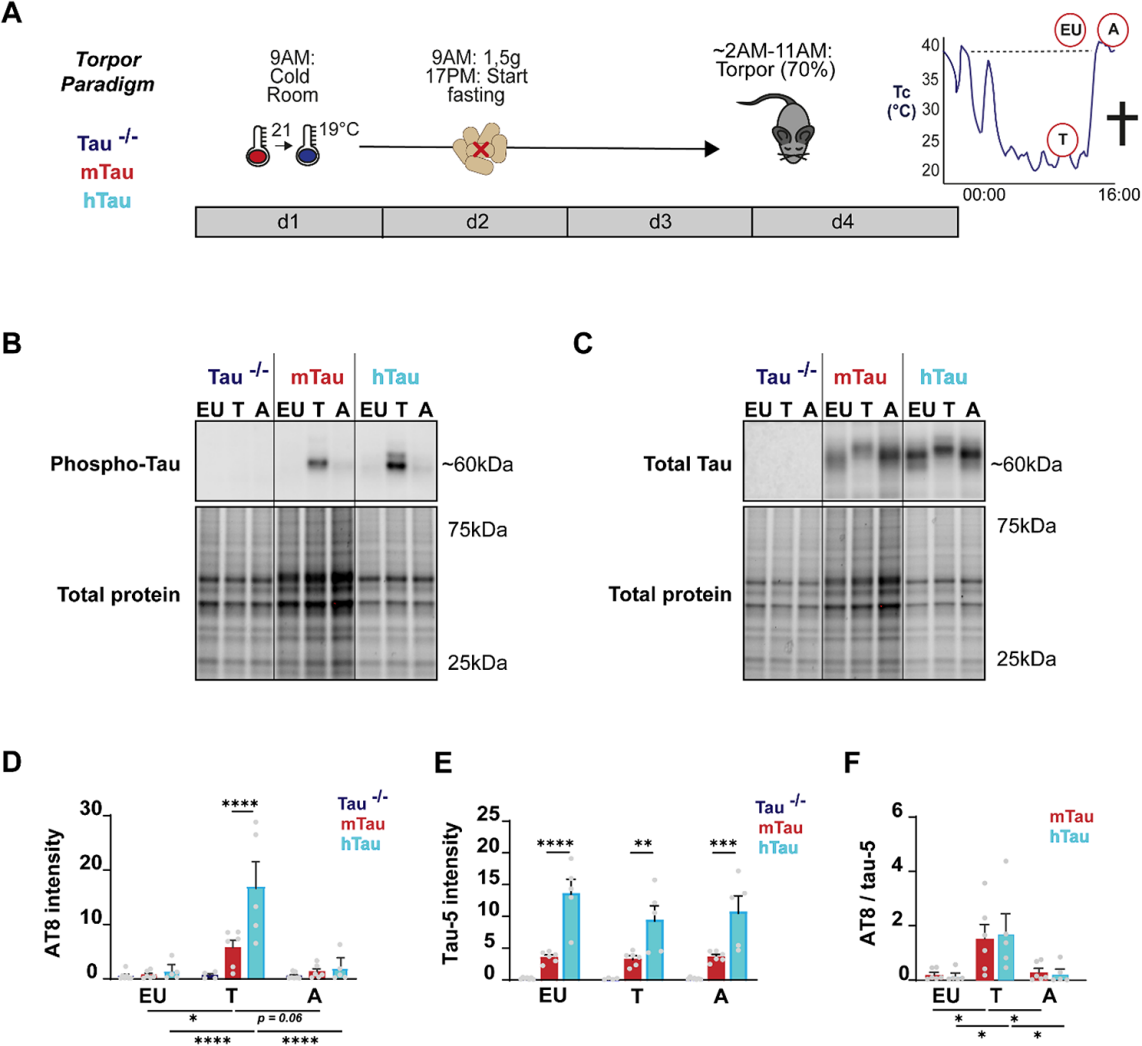
Phospho-tau and total tau levels during different torpor stages in the hippocampus of mtau and htau mice (**A**) Steady torpor was induced in mice using ambient temperature (T_a_) reduction on day 1 (from 21 °C to 19 °C) and food restriction on day 2 (1.5 g from 9AM till 17PM), followed by a fasting period of maximally 40 h. During the second night of fasting, ∼70% of the mice entered torpor [9]. Mice were euthanised during deep torpor (T; ∼10:00, T_b_ <26 °C for at least 6 h) or directly after arousal (A; ∼16:00, T_b_ 37 °C for 2 h). Euthermic (EU) control animals were held at normal housing temperature, fed ad lib, and euthanised on the same day ∼13:00. (**B**) Representative immunoblot images showing AT8 staining (Ser202/Thr205 hyperphosphorylated tau) in hippocampal lysates and gel images showing total protein loading. (**C**) Representative immunoblot images showing TAU-5 staining (total tau) in hippocampal lysates and gel images showing total protein loading. (**D**) Quantification indicates that AT8 intensity during torpor was significantly higher in mtau and htau mice compared to euthermic controls (5.63 ± 1.36 and 16.87 ± 4.50 vs. 0.63 ± 0.69 and 1.27 ± 1.70) and 3.0x higher in htau compared to mtau mice (2-way ANOVA, post-hoc Holm-Sidak, F_2,42_ = 12.67, **** *p* < 0.0001, *** *p* < 0.001, ** *p* < 0.01, * *p* < 0.05, ^n.s^. *p* > 0.05; tau^-/-^ EU: *n* = 7, T: *n* = 4, A: *n* = 7; mtau EU/T/A: *n* = 6; htau EU/T/A: *n* = 5). (**E**) Quantification shows that total tau levels do not differ between torpor stages, and that htau mice express ∼3x more total tau than mtau mice (htau EU: 13.67, T: 9.46 and A: 10.79; mtau EU: 3.63, T: 3.36 and A: 3.69; 2-way ANOVA, post-hoc Holm-Sidak, F_2,42_ = 65.0, **** *p* < 0.0001, *** *p* < 0.001, ** *p* < 0.01, * *p* < 0.05, ^n.s^. *p* > 0.05; tau^-/-^ EU: *n* = 7, T: *n* = 4, A: *n* = 7; mtau EU/T/A: *n* = 6; htau EU/T/A: *n* = 5). (**F**) Relative AT8 expression to TAU-5 levels shows similar AT8 levels in mtau and htau mice (htau EU: 0.169, T: 2.318 and A: 0.315; mtau EU: 0.223, T: 2.033 and A: 0.390; 2-way ANOVA, post-hoc Holm-Sidak, ^n.s^. *p* > 0.05)

We used C57Bl/6 wildtype mice expressing endogenous *MAPT* (referred to as mtau mice), *MAPT* knockout mice (referred to as tau^−/−^ mice), and *MAPT* knockout mice that uniquely express human tau isoforms (referred to as htau mice) under 40 h of fasting to induce steady and robust (T_b_ ≤ 26 °C for ≥ 6 h) torpor in ∼70% of the animals (Figure S1). We used body temperature as readout for the different phases of torpor, as others and we have previously shown that metabolism and body temperature are highly correlated [10, 16]. Hippocampal tissue was collected of mice terminated during torpor (in the cold phase) and during arousal (after rewarming), and of euthermic control mice (Fig. 1A). Phospho-tau levels were determined by immunoblotting using the AT8 monoclonal antibody that recognizes tau when phosphorylated at Ser202 and Thr205 [13, 17, 18] (Fig. 1B/D, S2). Phospho-tau levels were significantly higher in both mtau and htau mice during torpor compared to euthermia (*p* = 0.0490 and *p* < 0.0001, respectively), with ∼3.0x higher levels in htau mice compared to mtau mice (*p* < 0.0001). Tau hyperphosphorylation was completely reversed to baseline (euthermia) levels during arousal in both mtau and htau mice. Total tau levels, as measured by the TAU-5 monoclonal antibody [19], showed ∼3.2x higher tau expression in htau mice compared to mtau mice (Fig. 1C/E, S3; EU: *p* < 0.0001, T: *p* = 0.0016, A: *p* = 0.0003). However, total tau levels did not change during torpor in either mtau or htau mice. A mobility shift was observed for the total tau positive bands in all torpor samples, consistent with hyperphosphorylation during torpor. When AT8 intensity was corrected for total tau, AT8 levels were similar between mtau and htau mice, suggesting that higher phospho-tau levels in htau are due to higher expression of tau (Fig. 1F). As expected, no AT8 or TAU-5 reactivity was observed in Tau^−/−^ mouse (Fig. 1B-E, S2/3).

We confirmed tau hyperphosphorylation and reversion in mtau and htau mice using an additional phospho-epitope directed antibody that recognizes abnormally phosphorylated tau at Ser396 [20]. We found that similar to AT8, levels of Ser396-positive phosphorylated tau increased during torpor (EU vs. T: mtau *p* = 0.0613 and htau *p* = 0.0141) and were reversed upon arousal (EU vs. A: n.s.), substantiating the effect of torpor on pathological tau hyperphosphorylation and reversion (Figure S4-S5). Unlike AT8, Ser396 levels were already detectable in control animals (EU) and remained detectable at arousal both in mtau and htau mice. Importantly, fasted mtau mice that did not enter torpor (no torpor, NT) did not show increased levels of Ser396 phosphorylation. Together, our data show that torpor induces abnormal tau hyperphosphorylation as measured at two different phopho-epitopes (Ser202/THr205 and Ser396), and that this type of hyperphosphorylation is not induced by fasting only.

### Immunohistological analyses corroborates robust and reversible tau-hyperphosphorylation in multiple brain areas in mtau and htau mice

**Fig. 2:**
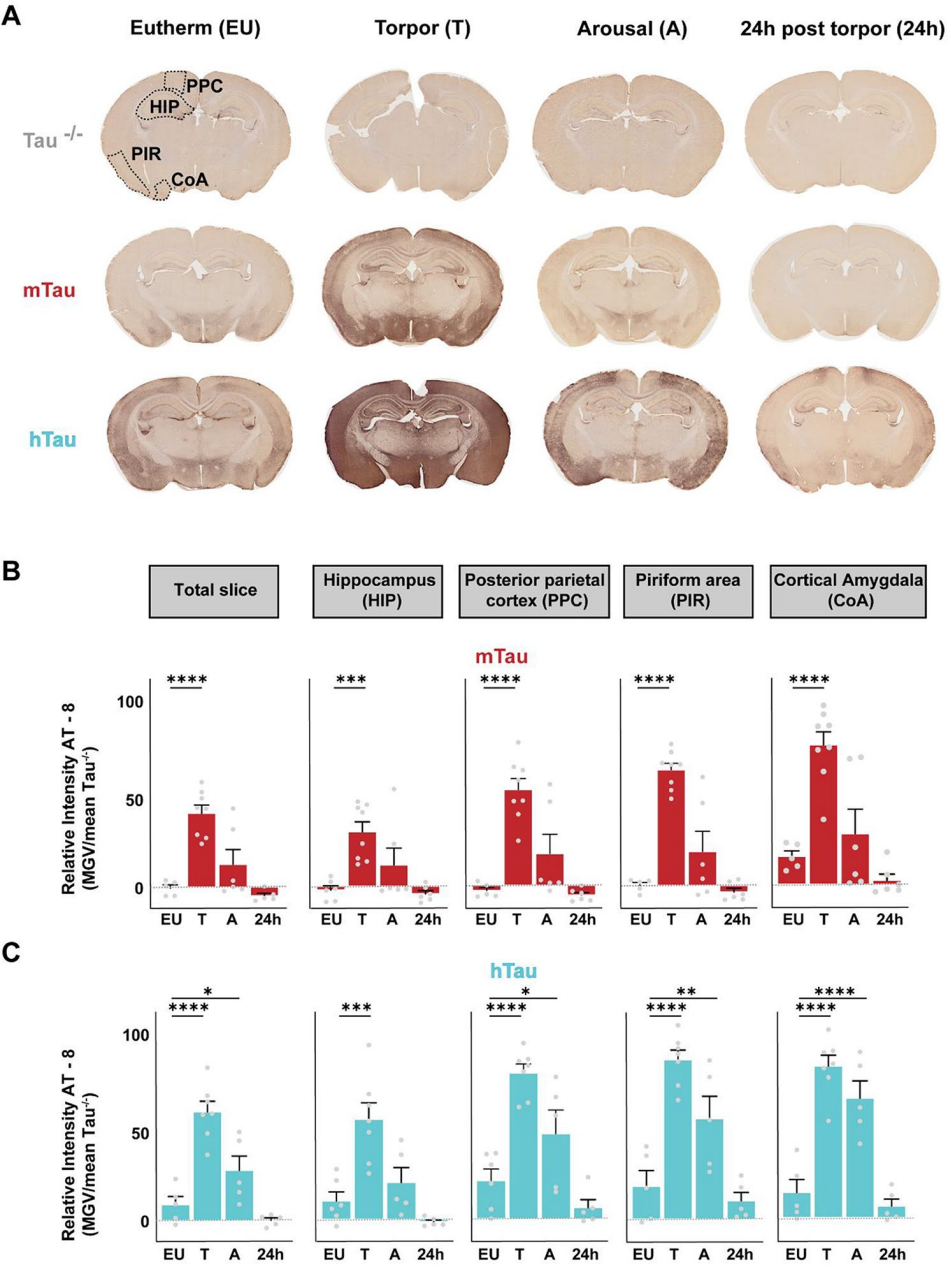
Immunohistological analysis of phospho-tau levels in mtau and htau brains (**A**) Representative coronal slices of eutherm (EU), torpor (T), arousal (A), and 24 post-torpor (24 h) tau^−/−^, mtau and htau mice, stained with AT8. Brain areas with high AT8 intensity were analysed separately (dashed lines): the hippocampus (HIP), posterior parietal cortex (PPC), piriform cortex (PIR) and cortical amygdala (CoA). (**B, C**) Quantification of AT8 intensity per area (corrected for mean tau^−/−^ mean gray value) shows a significant increase in tau phosphorylation during torpor for both mtau (**B**) and htau (**C**) mice in the total slice and all brain areas (EU vs. TL; *p* < < 0.05; mtau; total slice: t = 5.739, df = 20; HIP: t = 4.161, df = 22; PPC: t = 6.000, df = 21; PIR: t = 7.485, df = 21 and CoA: t = 5.235, df = 21; htau total slice: t = 6.673, df = 18; HIP: t = 4.744, df = 19; PPC: t = 6.006, df = 19; PIR: t = 6.463, df = 18 and CoA: t = 7.461, df = 18; 1-Way ANOVA, post-hoc Fisher’s LSD for all subsequent tests) which is reversed upon arousal for mtau mice (EU vs. A: *p* > 0.05) but not yet for htau mice where total slice, PPC, PIR and CoA AT8 intensity remains significantly higher during arousal (EU vs. A, *p* < 0.05; (total slice: t = 2.277, df = 18; PPC: t = 2.401, df = 19; PIRt = 2.197, df = 18 and CoA: t = 5.163, df = 18). At 24 h post-torpor, AT8 levels are completely reversed for all areas (EU vs. 24 h: *p* > 0.05; mtau total slice: t = 5.739, df = 20; HIP: t = 4.161, df = 22; PPC: t = 6.000, df = 21; PIR: t = 7.485, df = 21 and CoA: t = 5.235, df = 21; htau total slice: t = 6.673, df = 18; HIP: t = 4.744, df = 19; PPC: t = 6.006, df = 19; PIR: t = 6.463, df = 18 and CoA: t = 7.461, df = 18). 24 h after torpor phospho-tau was significantly lower than in EU for htau mice: 8.972 ± 4.525 vs. 0.1282 ± 1.391, HIP: 10.23 ± 4.919 vs. -1.379 ± 1.079, PPC: 21.21 ± 6.404 vs. 6.215 ± 4.410, PIR: 19.49 ± 9.239 vs. 10.98 ± 4.839 and CoA: 14.95 ± 7.625 vs. 6.962 ± 4.061; *p* < 0.05 for total slice, HIP and PPC, unpaired student’s t-test. * *p* < 0.05, ** *p* < 0.01, *** *p* < 0.001, **** *p* < 0.0001)

Next, we measured phospho-tau levels in coronal slices (∼2.33 mm from bregma) obtained in euthermia, during torpor, during arousal or 24 h post-torpor in tau ^−/−^, mtau and htau mice using AT8 staining (Fig. 2A). Mean phospho-tau intensity was determined in the total slice, and in four separate brain regions with the strongest AT8 staining in the posterior parietal cortex (PPC), the hippocampus (HIP), the piriform cortex (PIR) and cortical amygdala (CoA), when corrected for mean intensity measured in tau^−/−^ mice (negative control). All areas measured were of comparable size between groups (Figure S6; for all ^n.s^*p* > 0.05).

Torpor significantly increased AT8 staining compared to euthermia in the total slice and in all four brain regions in both mtau and htau mice (Fig. 2B/C; EU vs. T; mtau; total slice: *p* < 0.0001; HIP: *p* < 0.0004, PPC: *p* < 0.0001, PIR: *p* < 0.0001 and CoA: *p* < 0.0001; htau total slice: *p* < 0.0001, HIP: *p* = 0.0001, PPC: *p* < 0.0001, PIR: *p* < 0.0001, and CoA: *p* < 0.0001). Upon arousal, phospho-tau was reversed back to baseline for mtau mice (EU vs. A; *p* > 0.05 for all), but remained significanlty higher than EU in htau mice (EU vs. A: total slice: *p* = 0.0352, PPC: *p* = 0.0268, PIR: *p* = 0.0050, and CoA: *p* < 0.0001). At 24 h after torpor, phospho-tau intensities in htau mice were also reversed back to baseline (EU) and tau phosphorylation even appears to be lower than baseline for all areas (EU vs. 24 h total slice; total slice: *p* = 0.0439, HIP: *p* = 0.0325, PPC: *p* = 0.0490, PIR: *p* = 0.2191, CoA: *p* = 0.1911). As expected, tau^−/−^ slices did not show any phospho-tau staining (Figure S7).

To confirm these findings, an additional phospho-epitope directed antibody that recognizes abnormally phosphorylated PHF tau (AT100, directed against Thr212 and Ser214) [21] was used (Figure S9). We found that similar to AT8, levels of AT100-positive phosphorylated tau were significantly increased during torpor in htau mice (EU vs. T: *p* = 0.0248) and were reversed upon arousal (T vs. A: *p* = 0.0029), substantiating the effect of torpor on pathological tau hyperphosphorylation and reversion. Furthermore, AT100 immunoreactivity, similar to AT8, was lower at 24 h after torpor than at EU. Together our data demonstrate robust yet reversible hyperphosphorylation of tau in multiple brain areas and using multiple antibodies and indicate a potential clearance of pre-existing phospho-tau after a single torpor bout in htau mice.

### Reversible somato-dendritic accumulation of hyperphosphorylated tau in htau mice

**Fig. 3:**
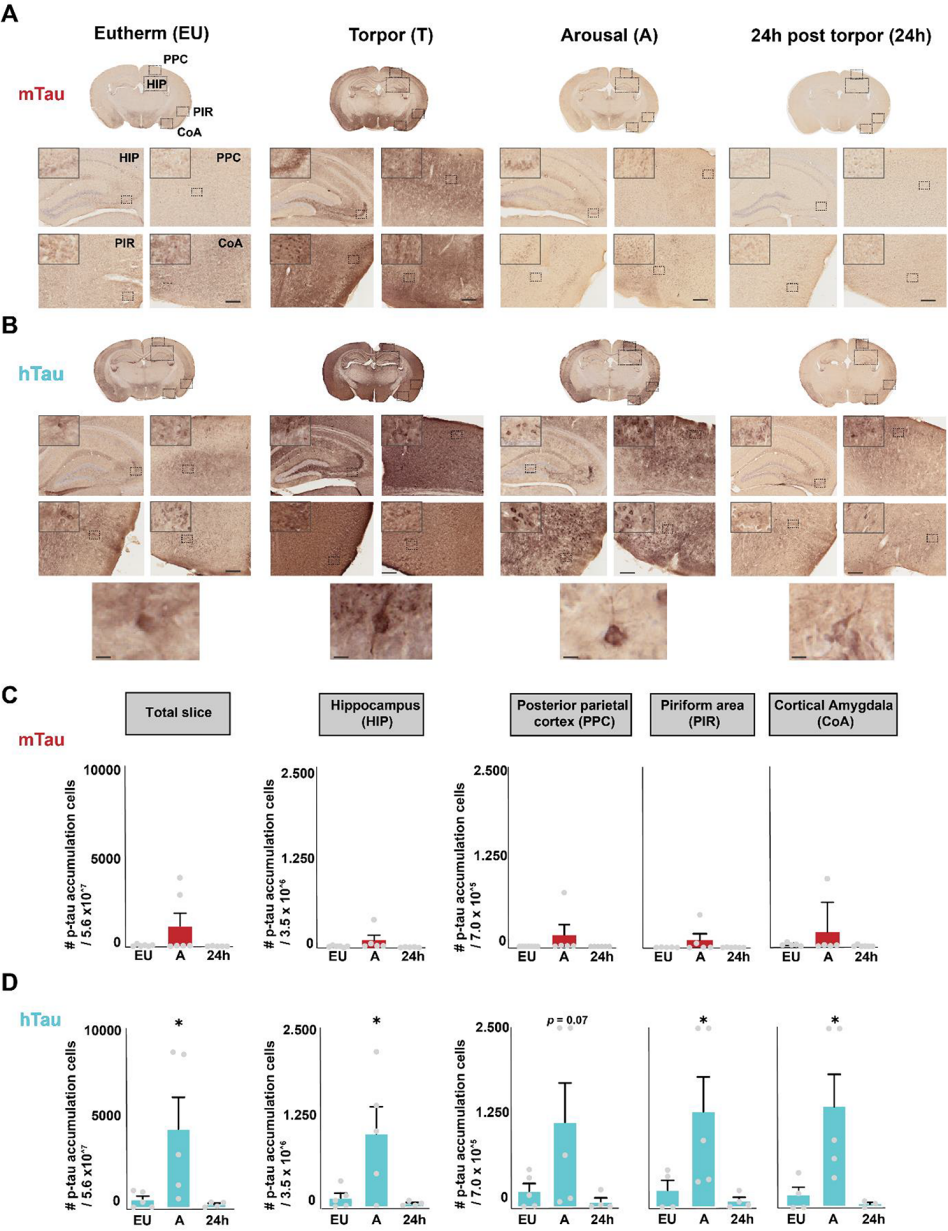
Cells with accumulated p-tau in mtau and htau brains (**A, B**) Representative total coronal slices and zoom-ins of the PPC, HIP, PIR and CoA for (**A**) mtau and (**B**) htau EU, T, A and 24 h post-torpor mice. Zoom-ins reveal diffuse AT8 staining in all torpor stages in mtau mice, whereas in htau mice EU, T, A and 24 h post-torpor mice show cells with p-tau accumulation in all four brain areas. Upper left corners show extra zoom-ins of the indicated areas. Below are zoom-ins of somato-dendritic accumulation of phosphorylated tau in hippocampus that are observed in htau mice specifically in all phases of daily torpor. Scale bars represent 200 and 20 μm. (**C, D**) The number of cells with p-tau accumulation per brain area (the same size of the brain area was measured for every animal; 5.6 × 10^7^ for total slice, 3.5 × 10^6^ for HIP and 7,0 × 10^5^ for PPC, PIR and CoA) was determined in EU, A and 24 h post-torpor (note that cells with p-tau accumulation could not be measured during torpor due to high AT8 intensities) for PPC, HIP, PIR and CoA of (**C**) mtau mice and (**D**) htau mice. Cells with p-tau accumulation were low in mtau mice and not significantly higher during arousal (EU vs. A, *p* > 0.05 for all, total slice: F_2, 14_ = 2.179, HIP F_2, 13_ = 1.845, PPC F_2, 15_ = 1.503, PIR: F_2, 13_ = 1.472 and CoA: F_2, 13_ = 1.0771-Way ANOVA). In htau mice on the other hand, cells with p-tau accumulation already appeared in EU and were significantly higher during arousal for total slice, HIP, PIR and CoA (EU vs. A; *p* < 0.05, total slice: t = 2.595, df = 11, HIP: t = 2.777, df = 11, PPC: t = 2.008, df = 11, PIR: t = 2.315, df = 10 and CoA: t = 2.673, df = 9; 1-Way ANOVA, for all areas). 24 h after torpor the number of cells with p-tau accumulation reversed back to euthermic levels (*p* > 0.05; 1-Way ANOVA; total slice: t = 0.1651, df = 11; HIP: t = 0.2326, df = 11; PPC: t = 0.2995, df = 11; PIR: t = 0.3026, df = 10; and CoA: df = 9, t = 0.2326). Though not significant, levels of cells with p-tau accumulation appeared lower at 24 h vs. A in htau mice (total slice: 2588 ± 2030 vs. 1922 ± 710.1, HIP: 139.3 ± 65.4 vs. 49.5 ± 16.2, PPC: 223.3 ± 103.9 vs. 71.4 ± 57.4, PIR: 240.4 ± 137.4 vs.89.4 ± 51.2 and CoA: 172.3 ± 114.4 vs. 49.8 ±. 24.4). *p < 0.05

To assess tau accumulation in mtau and htau mice we quantified cells with somato-dendritically localized AT8-reactive tau (p-tau), as defined in [13–15], in zoomed images of AT8-stained PPC, HIP, PIR and CoA (Fig. 3A, B, Figure S8). Tau hyper-phosphorylation was primarily diffuse in mtau mice throughout all phases (EU, T, A, 24 h), whereas in htau mice cells with AT8-reactive accumulation of tau were observed in all phases. These cells with accumulated p-tau were almost absent in euthermic mtau mice and no significant increase in cells with accumulated p-tau was observed for any of the measured areas during arousal (Fig. 3A/C, EU vs. A, total slice: *p* = 0.150, HIP: *p* = 0.197, PPC: *p* = 0.2541, PIR: *p* = 0.2712 and CoA: *p* = 0.3692; note that cells with accumulated p-tau were not assessed during torpor due to intense AT8 staining). In htau mice, cells with accumulated p-tau were already present in euthermic animals (Fig. 3B/D). A significant increase in cells with accumulated p-tau was observed during arousal in all four brain areas (EU vs. A; total slice: *p* = 0.0249, HIP: *p* = 0.0180, PPC: *p* = 0.0698, PIR: *p* = 0.0431 and CoA: *p* = 0.0255). Interestingly, the amount of cells with accumulated p-tau was completely reversed at 24 h post-torpor, with the number of cells with accumulated p-tau not significantly higher than baseline (EU) levels (24 h vs. A, *p* > 0.05 for all areas). Similar to the total AT8 intensity, the number of cells with accumulated p-tau at 24 h post-torpor appears to be even lower for all areas compared to baseline EU, though not significant (EU vs. 24 h; *p* > 0.05 for all areas). Taken together, these data show that htau mice have low levels of cells with accumulated p-tau before torpor induction. The number of cells with accumulated p-tau increases significantly at arousal, and disappear after torpor with numbers dropping below baseline at 24 h after torpor.

## Discussion

We established that daily torpor, similar to seasonal hibernators, induces robust and reversible tau phosphorylation in the brains of mice. In addition, we made use of this model to study tau phosphorylation behaviour of human tau instead of native tau. The observed phosphorylation was strongest in the hippocampus, posterior parietal cortex, piriform cortex and cortical amygdala. While AT8 staining was mostly diffuse in mtau mice, htau mice also expressed somato-dendriric accumulation of hyperphosphorylated tau resembling pre-tangles observed in Alzheimer brain throughout all stages of torpor and in all brain areas. Both phosphorylated tau levels and the appearance of cells with accumulated p-tau were significantly reduced at 24 h after torpor reaching levels that, depending on brain area, were comparable to or even lower than baseline, indicating that tau dephosphorylation and/or clearance mechanisms are prompted during arousal. This study demonstrates that daily torpor in mice offers a quick, controllable and standardized method to uniquely study tau phosphorylation behaviour of human tau.

The htau mouse line used in this study, expresses non-mutated human tau, and was generated by crossing *Mapt*^−/−^ mice with 8 C founder mice expressing a human *MAPT* transgene [22]. An altered ratio of tau isoforms favouring 3R tau is detected in this model due to the loss of mtau, which is predominantly 4R tau. Htau mice are reported to display tau accumulation from 6 months of age, as shown by somato-dendritic accumulation of phosphorylated tau, and tau pathology was observed at 9 months of age as shown by higher levels of sarkosyl insoluble tau and electron microscopic detection of paired helical filaments [22, 23]. Our observations indicate cells with somato-dendritic accumulation of phosphorylated tau already appear in 3-months-old euthermic htau mice. Interestingly, even though similar levels of htau are also present in the 8 C founder line, containing both mtau and htau, no tau aggregation was observed in this line, suggesting that mtau has a dominant protective role over htau in these mice, possibly due to a higher levels of 4R tau [24]. Indeed, torpor induces only small amounts of cells with somato-dendritic accumulation of AT8 in mtau mice, whereas in htau mice cells with accumulated p-tau are present in all brain areas examined, even at baseline level. Although we cannot exclude that the 3x higher AT8 and total tau levels in htau mice may also have contributed to the excess of phosphorylated tau (p-tau) accumulation in htau mice, the almost all-or-none difference in the number of cells with p-tau positive accumulation that remain present in arousal only in htau and not in mtau mice seems to suggest differences in p-tau accumulation and reversion mechanisms, although the higher tau expression levels in htau mice needs to be taken into account. This should be investigated further in mouse lines that express human tau at comparable levels. Tau phosphorylation and hyperphosphorylation are normal homeostatic physiological mechanisms in neurons which are tightly regulated by tau kinases and phosphatases [25]. During torpor, these physiological mechanisms are thought to be employed to protect neurons from excitotoxicity by altering neuronal plasticity [26, 27]. Tau hyperphosphorylation during torpor results from an additional suppression of phosphatase activity (e.g. PP2A) and increase in kinase activity (e.g. cdk5) upon lowering of temperature [28]. In addition, torpor-associated tau hyperphosphorylation might be regulated via reciprocal glucose-dependent regulation of O-GlcNAcylation [29, 30] and by activation of the unfolded protein response, which confers metabolic stress-induced tau hyperphosphorylation [31]. Therefore, hibernation-associated hyperphosphorylation of tau seems to be a natural process that differs from the passive hypothermia-associated increase in tau hyperphosphorylation due to decreased phosphatase activity alone. Interestingly, these same pathways have also been suggested to be involved in AD-associated tau hyperphosphorylation [32]. No torpor (NT) samples of mtau mice that were fasted but did not enter torpor, did not shown similar tau hyperphosphorylation levels as was observed in mtau torpor mice (Figure S4), substantiating that tau hyperphosphorylation is a torpor-induced process. This is in line with previous research showing that torpor can be prompted without fasting when QRFP-expressing neurons in the preoptic area of the hypothalamus or hypothalamus ADCYAP1-positive neurons are stimulated [12, 33]. Regulation of tau hyperphosphorylation during torpor in seasonal hibernators is thought to be essential as it dissociates tau from microtubules, leading to dendritic retraction and priming structural plasticity in neurons necessary during arousal [26, 27]. As this physically also prevents neuronal communication, it is thought to avert glutamate-induced excitotoxicity during torpor [26, 34]. We previously showed that daily torpor in mice does not lead to big structural neuronal rearrangements, but rather to an altered synaptic molecular composition, and it is therefore not likely that the observed tau hyperphosphorylation during torpor leads to structural regulation of neuronal communication [10]. Another protective pathway by which tau hyperphosphorylation could prevent neuronal communication and glutamate excitotoxicity during daily torpor is the recently discovered blockade of presynaptic vesicle release via binding of hyperphosphorylated tau to synaptogyrin-3 [35]. The notion that tau knockout mice were capable of entering and exiting torpor in this study seems to argue against an important protective role for tau hyperphosphorylation during torpor, however potential post-hibernation damage resulting from excitotoxicity or impact on post-hibernation plasticity were not assessed in these animals.

In contrast to the physiological tau (hyper)phosphorylation prompted by torpor to induce neuroplasticity and/or protect against excitotoxicity, tau aggregation in neurodegenerative diseases constitutes pathological dysfunctional protein accumulation which is only counteracted by proteasomal or autophagy–lysosomal clearance [36]. Pathological accumulation of tau would not be favorable during torpor and indeed we did not observe any significant cells with accumulated p-tau in mtau mice during or after torpor. In htau mice, on the other hand, significant levels of accumulated phospho-tau were observed. This htau p-tau accumulation, however, was completely reversable upon arousal, indicating that mice uniquely employ torpor-induced clearance mechanisms to get rid of accumulated p-tau either by removing phosphorylation at these AD specific phosphorylation sites (Ser202 and Thr205: AT8) or by removing the p-tau itself, both of which does not seem to happen in Alzheimer patient brain. It should be noted that although AT8 detects phosphorylation at two well-known tau phosphorylation sites and is commonly used for post-mortem biochemical staging of AD [13], it does not specifically detect mature tau tangles, but also stains pre-tangles in human brain, which appear as somato-dendritic accumulation of AT8 positive tau [37]. Additional staining methods, such as PHF-1 and Tau-C3 of other p-tau epitopes, could therefore shed light on the tangle maturity of the somato-dendritic accumulation of AT8 observed during torpor. Previous studies indeed found paired helical filaments in htau mice, and demonstrating the reversibility of paired helical filaments specifically would be valuable and of importance as the stage of accumulation may influence the reversibility of the process and subsequent mechanisms of clearance [38]. Future experiments should focus on identifying the nature of torpor-induced tau hyperphosphorylation and accumulation in htau mice, for example by measuring phospho-epitopes in soluble vs. insoluble fractions. In addition to tau tangles, AD is also characterized by pathological amyloid-beta aggregates. It is still unclear how tau and amyloid-beta pathogenically interact [32]. Daily torpor in mice may offer unique opportunities to study this interaction in the future, for example by inducing torpor in hTau mice with or without pathogenic mutations in the amyloid precursor protein (APP), preferably in a crossbreed that expresses htau at more physiological levels [39].

In this study, we also identified four brain areas that are particularly affected by torpor-induced tau hyperphosphorylation and accumulation, i.e., the hippocampus, the posterior parietal cortex, the piriform cortex and the cortical amygdala. Similar to Braak staging of AD, tau in the hippocampus is highly phosphorylated in torpor mice [13], especially in the CA3 and dentate gyrus (DG). Surprisingly, the DG was not reactive in ground squirrel upon torpor and arousal [9], indicating potential interspecies differences in brain areas affected by torpor. The posterior parietal cortex, which was also highly reactive in our experiments, has been shown to be affected early in AD [40] and is highly connected to the hippocampus [41]. The piriform cortex, of which disruption leads to early olfactory deficits in AD [42], was also highly reactive to AT8. This is in line with a recent study showing that hibernation in golden hamster disrupts odor recall [6]. Finally, the cortical amygdala was severely affected. Post-mortem and neuroimaging studies have established the amygdala as one of the earliest sites of tau aggregation in AD, which is thought to cause secondary disease outcomes such as anxiety [43, 44]. Although it is of interest to observe that torpor and AD share brain regions vulnerable to tau hyperphosphorylation, future experiments should investigate the face validity of the torpor model in relation to AD-associated tau pathology.

Our data demonstrate that daily torpor in mice can be used to study tau phosphorylation from both a physiological as well as a (human) pathophysiological perspective. Moreover, mice seem to deploy p-tau dephosphorylation and/or clearance pathways that facilitate the removal of human phosphorylated tau accumulation during arousal, making torpor a very attractive model to discover novel treatments for tauopathies like AD. While previous work already established the reversibility of torpor-induced tau hyperphosphorylation in seasonal hibernators, these studies are limited by the fact that only endogenously expressed tau was examined, thus not reflecting AD-relevant human tau pathology. We propose that daily torpor in AD-relevant transgenic mouse models (e.g., htau mice with or without amyloid pathology) can be used to better model processes involved in human tau pathology, with the prospect to uncover disease-relevant accumulation and clearance mechanisms and potential intervention targets therein.

## Methods

### Animals

All experiments with animals were approved by the local animal research committee (IVD) and complied with the national central committee of animal research (CCD #15167) ordination. C57BL/6J (*stock number: 000664|Black6, Jackson)* for mTau mice and B6.Cg-*Mapt*^*tm1(EGFP)Klt*^ Tg(MAPT)8cPdav/J (*stock number: 005491|htau, Jackson)* for *t*^-/-^ - and hTau mice, derived from the same strain, were bred locally in the Amsterdam Animal Research Center (AARC). Male animals were used in all experiments. All mice were 3 months of age +/- 1 week. Mice were single-housed on sawdust in standard Makrolon type 2 cages (∼21 °C ambient temperature (Ta) and ∼50% humidity), enriched with cardboard nesting material and chewing wood and with food and water ad lib. Mice were kept on a 12:12 light-dark cycle, with lights on at 7:00 AM.

### Temperature logger implantation

Animals were injected s.c. with 0.05 mg/kg buprenorfine 30 min prior to surgery. Real-time readable temperature loggers (Anipill; Animals Monitoring, Hérouville, France) were implanted intra-peritoneally under full anesthesia (1.5-3% isoflurane in oxygen). Post-operation analgesia (buprenorfine 0.05 mg/kg) was provided. Animals were allowed to recover from surgery for at least 1 week before the start of the torpor paradigm.

### Torpor paradigm

The torpor paradigm has been published previously [10]. In short: on day 1, animals were housed at a T_a_ of 19 °C. On day 2, fasting was started at 17:00. Mice typically entered torpor the second night of fasting of the paradigm. Mice were euthanized at the following time points: *day 4* ∼10:00 (torpor group; T), ∼16:00 (arousal group; A), *day 5* ∼13:00 (24 h post torpor group; 24 h). All T, A and 24 h mice underwent a torpor bout of at least 6 h with a T_b_ <26 °C. Euthermic (EU) control mice were maintained on food ad libitum at a T_a_ of ∼21 °C and sacrificed at ∼13:00 on day 4 of the torpor paradigm. Mice that lowered T_b_ less than 6 h or showed highly oscillating patterns (30%), were removed from the experiments. All mice were euthanized using decapitation for molecular analysis or perfusion under tribromoethanol anesthesia for immunohistochemical analyses. The brain was processed as detailed below.

### Immunoblotting

Hippocampal tissue was collected from Tau^−/−^ mice: euthermic (EU; *n* = 7), torpor (T; *n* = 4), arousal (A; *n* = 7); mTau mice: EU *n* = 6, T *n* = 6, A *n* = 6; and hTau mice: EU *n* = 5, TL *n* = 5, AL *n* = 5. The hippocampal tissue was homogenized in ice-cold homogenization buffer (0.32 M sucrose, 5 mM HEPES/NaOH, PH 7.4) containing 1 tablet of cOmplete™ EDTA-free Protease Inhibitor Cocktail per 50 mL (Sigma-Aaldrich) and 1 tablet of PhosStop per 10 mL (Sigma-Aaldrich). The homogenate was then centrifuged at 1,000 g for 10 min at 4 °C after which the supernatant was collected.

SDS sample-buffer (Laemmli) was added and samples were heated to 96 °C for 5 min. 10 µg sample was loaded on a Criterion TGX stain-free gel (BioRad). Total protein loading was determined using protein activation imaging (Gel-Doc EZ system, BioRad) and analyses with ImageLab 6.0.1 (BioRad). After transfer onto a PVDF membrane (overnight, 40 V, 4 °C), blots were incubated with primary antibodies against AT8 (Thermofischer, Ser202/Thr205, 1:500) and Tau-5 (Thermofischer, anti-tau, 1:500) overnight at 4 °C, and subsequently with secondary antibody (Sigma-Aaldrich, Gt-antiMS HRP, 1:10.000) 2 h at RT. Blots were scanned with an Odyssey Fc System (Li-Cor) after ECL incubation (2 min) and analyzed using ImageStudio Lite 5.3 (Li-Cor). Total protein loading was used for normalization.

### Immunohistochemistry

After animals were sacrificed, brains were perfused and post-fixated in 4% PFA overnight, followed by two days in 30% sucrose. Within two weeks 35 μm coronal slices of tau ^−/−^ brain: EU (*n* = 4), T (*n* = 5), A (*n* = 4), 24 h (*n* = 3); mTau brain: (EU; *n* = 6), torpor (T; *n* = 8), arousal (A; *n* = 6), 24 h (*n* = 6); and hTau brain: EU (*n* = 6), *t* (*n* = 7), A (*n* = 5), 24 h (*n* = 5) were made using a cryostat (-15 °C; Leica) and kept in PBS + azide. The sections were washed in phosphate-buffered saline (PBS; 1x, pH 7.4) at room temperature, and one slice per animal (∼2.3 mm from bregma) was incubated with blocking solution [ 1x PBS, Normal goat serum (5%), 2,5% BSA, 0,2% Triton], for an hour, and primary antibody AT8 (Thermofischer, Ser202/Thr205, 1:400) diluted in blocking solution, overnight. Between every incubation step the sections were washed three times ten minutes. The slices were probed with goat anti-mouse HRP (Sigma-Aaldrich, Gt-antiMS HRP, 1:100) for one hour and incubated with DAB (3,3′-Diaminobenzidine) for three minutes. Once mounted and dried, the sections were counterstained in Hematoxylin (Mayer’s Hemalum solution; Merck) for three minutes and dehydrated, followed by one minute in Xylene and cementing with Entellan (Merck).

Sections were scanned on a Vectra Polaris whole-slide scanner (Akoya Biosciences) using a 20x objective and scans were analysed using QuPath-0.3.2 and Fiji [45, 46]. Images were inverted and total slice, PPC, HIP, PIR or CoA was selected and measured. Intensities (mean gray value) were determined for the 2 mirroring brain areas and the mean was used for further statistical analyses, so that one datapoint represented one animal. Tau -/- MGV was used for background subtraction. When brain areas were broken or when the resolution in a brain area was too low, they were not included in the intensity analyses or tau accumulation score analyses respectively, hence the difference in numbers of slices and brain areas measured per group. The same holds for the analyses of cells with p-tau accumulation in Fiji using a pre-set threshold and the analyze particles tool (Figure S8).

### Data analysis and statistics

GraphPad Prism 8.02 (for Windows, GraphPad Software, La Jolla, CA) was used for statistical analyses. For all statistical tests, a *p* < 0.05 was considered significant. Error bars show the standard error of the mean (SEM). The number of animals used for statistical analysis are indicated in all graph legends, and by showing all data points. 2-way ANOVA was used with a post-hoc Holm-Sidak test or a 1-Way ANOVA with post-hoc Fishers’ LSD was used for multiple comparisons. Single statistical comparisons were performed using Student’s *t*-test. Samples were excluded from analyses when slices or brain areas were broken or when the resolution was insufficient.

### Electronic supplementary material

Below is the link to the electronic supplementary material.

Supplementary Material 1

## Data Availability

All data is available, either as supplemental data set of this manuscript, or by request to the corresponding authors.
