# Supplementary material for: Torpor induces reversible tau hyperphosphorylation and accumulation in mice expressing human tau
Source: Acta Neuropathol Commun. 2024 Jun 4;12:86. doi: 10.1186/s40478-024-01800-4 (PMC11149198; doi:10.1186/s40478-024-01800-4)
Supplement: Supplementary file 1 — Supplementary Material 1 [file 40478_2024_1800_MOESM1_ESM.docx]

#
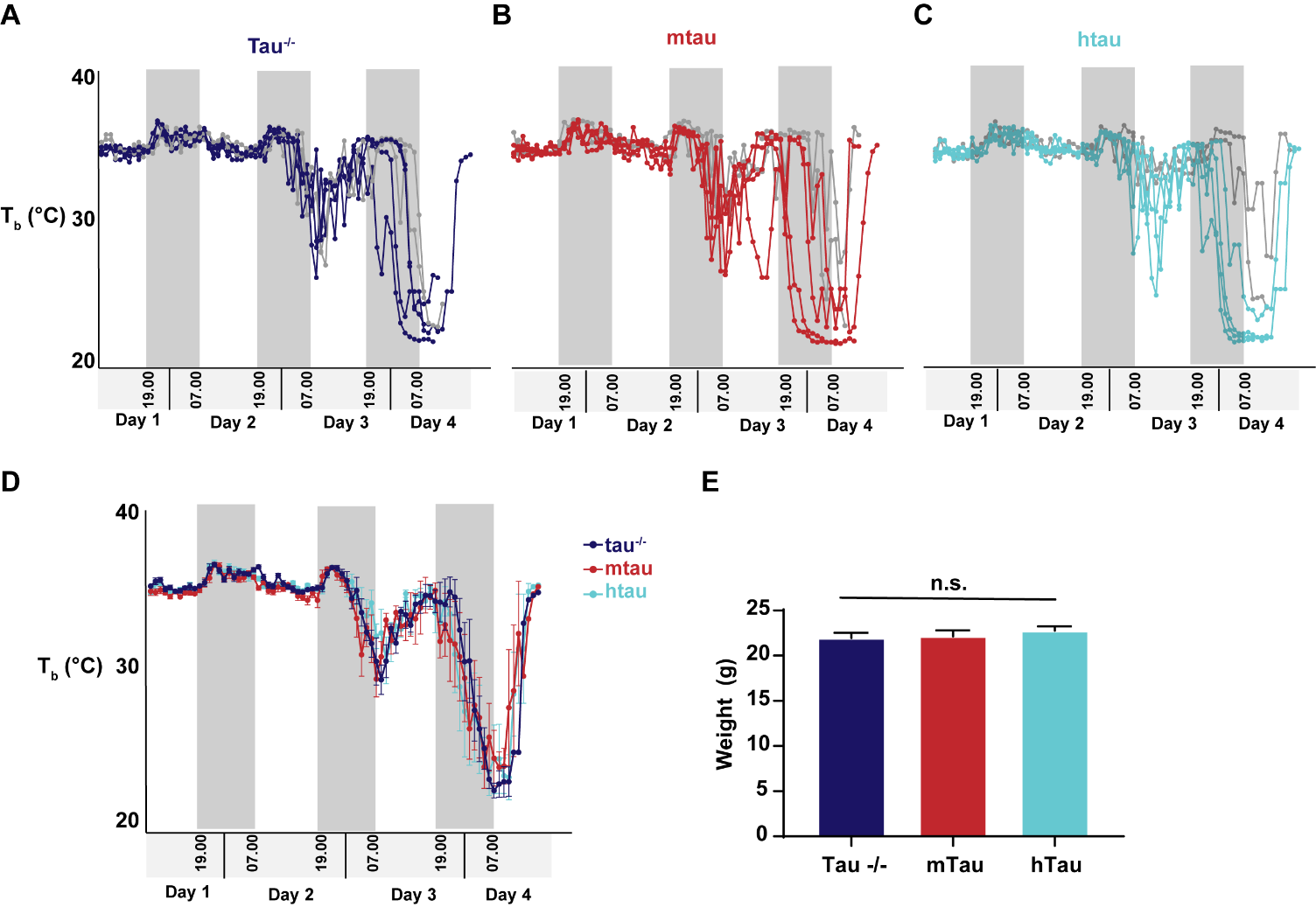
Supplementary data

**Figure S1: Temperature logger data and weights of one batch Tau^-/-^, mtau and htau mice shows no difference in torpor success and weight**

(**A-C**) Temperature logger data of Tau -/-, mtau and htau mice of 1 batch (n = 6 / group). Samples that were included (steady and robust torpor; T_b_ ≤ 26, ≥6h) are shown in color, samples that were excluded based on these inclusion criteria are shown in grey. (D) Mean temperature and standard error of the mean of the 6 animals per group shows no difference in torpor induction and success in the 3 groups. (E) the start weight of the three groups was the same (One-way ANOVA, F_2, 15_ = 0.543, *p* = 0.597).


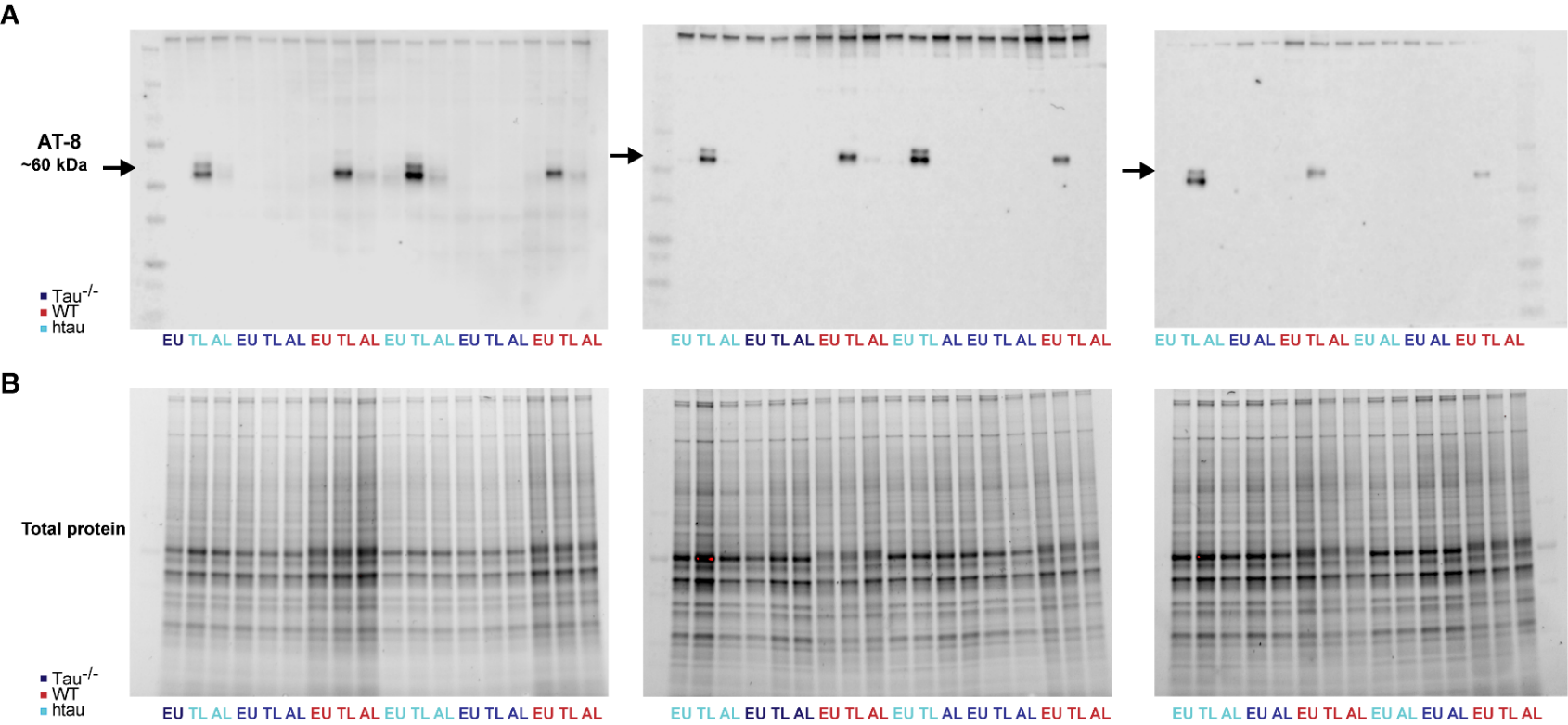


### Figure S2: AT8 immunoblots and corresponding gels

(**A**) AT8 immunoblots for all Tau -/- (dark blue), mTau (red) and hTau (light blue) samples showed bands around 60kDa; indicated with arrow. (**B**) ECL protein staining of total protein levels was used for normalization.

###
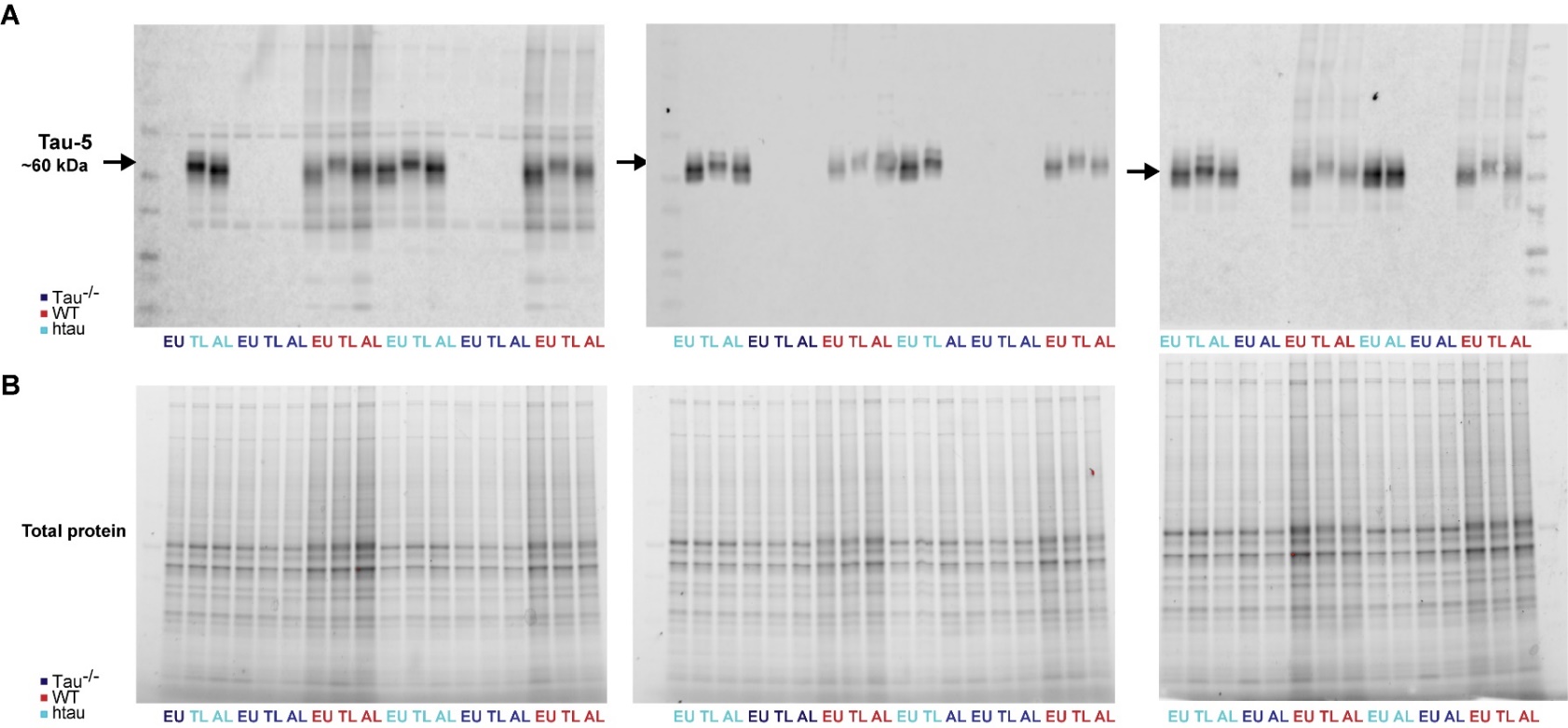


### Figure S3: Tau-5 immunoblots and corresponding gels

(**A**) Tau-5 (total Tau) immunoblots for all Tau -/- (dark blue), mTau (red) and hTau (light blue) samples showed bands around 60kDa; indicated with arrow. (**B**) ECL protein staining of total protein levels was used for normalization.

###
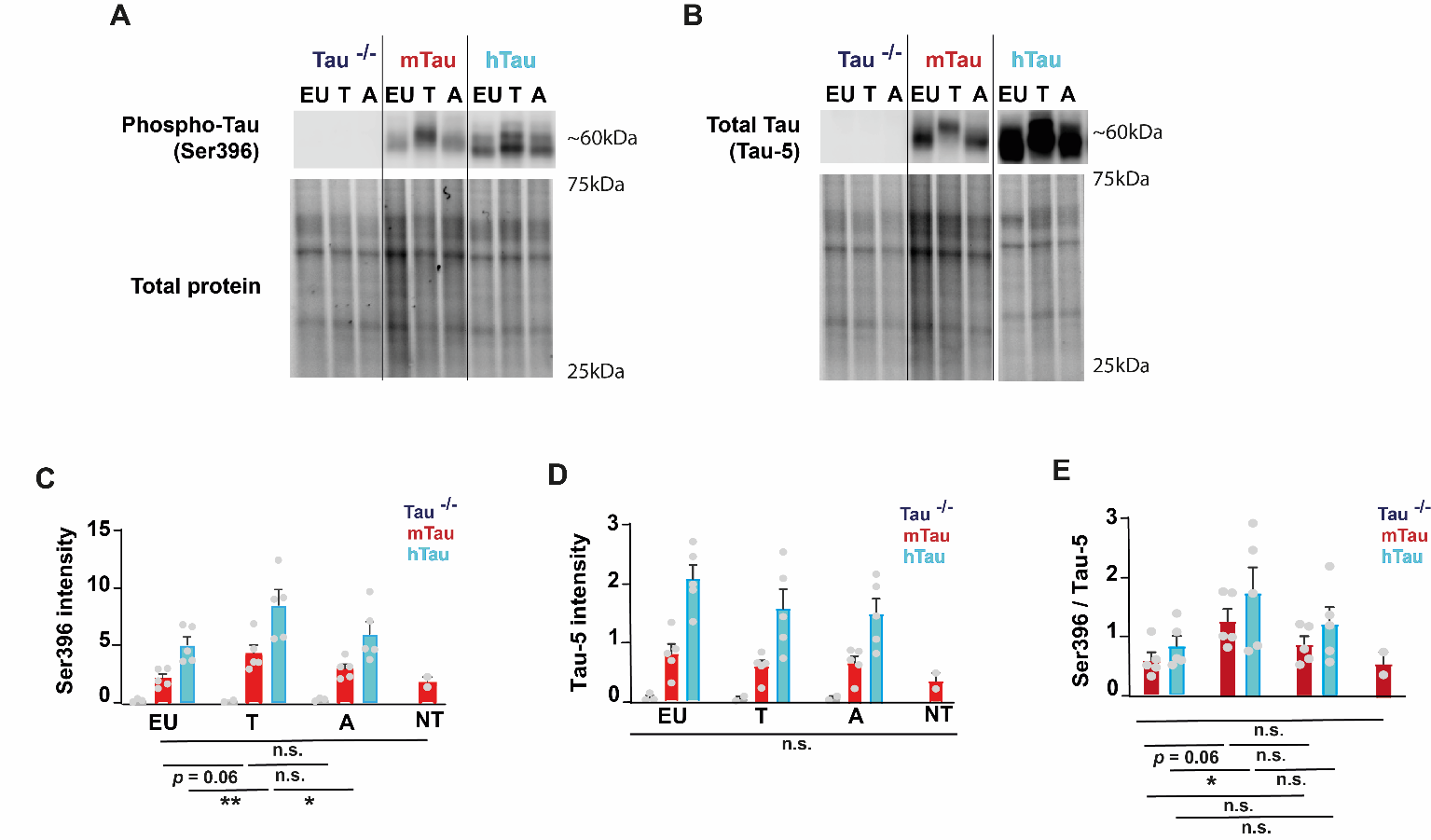


### Figure S4: Phospho-tau (ser396) and total tau levels during different torpor stages in the hippocampus of mtau and htau mice

(**A**) Representative immunoblot images showing Ser396 staining (hyperphosphorylated tau) in hippocampal lysates and gel images showing total protein loading. (**B**) Representative immunoblot images showing TAU-5 staining (total tau) in hippocampal lysates and gel images showing total protein loading. (**C**) Quantification indicates that Ser396 intensity during torpor was higher in mtau and htau mice compared to euthermic controls (4.111 ± 1.517 and 8.157 ± 2.962 vs. 2.012 ± 0.759 and 4.814 ± 1.509|) and 2.0x higher in htau compared to mtau mice (2-way ANOVA, post-hoc Holm-Sidak, F_2,29_ = 36,69 , ** *p* < 0.01, * *p* < 0.05, ^n.s.^ *p* > 0.05; tau^-/-^ EU: n = 3, T: n = 2, A: n = 3; mtau EU/T/A: n = 5; htau EU/T/A: n = 5). (**D**) Quantification shows that total tau levels do not differ between torpor stages, and that htau mice express ~3x more total tau than mtau mice (htau EU: 2.107, T: 1.549 and A: 1.481; mtau EU: 0.776, T: 0.587 and A: 0.666; 2-way ANOVA, post-hoc Holm-Sidak, F_2,28_ = 37.68, ^n.s.^ *p* > 0.05; tau^-/-^ EU: n = 3, T: n = 2, A: n = 2; mtau EU/T/A: n = 5; htau EU/T/A: n = 5). (**E**) Relative Ser396 expression to TAU-5 levels shows more similar Ser396 levels in mtau and htau mice (htau EU: 1.684, T: 3.613 and A: 2.476; mtau EU: 1.188, T: 2.618 and A: 1.750; 2-way ANOVA, post-hoc Holm-Sidak, F_1,24_ = 3.090, * *p* < 0.05, ^n.s.^ *p* > 0.05).

**
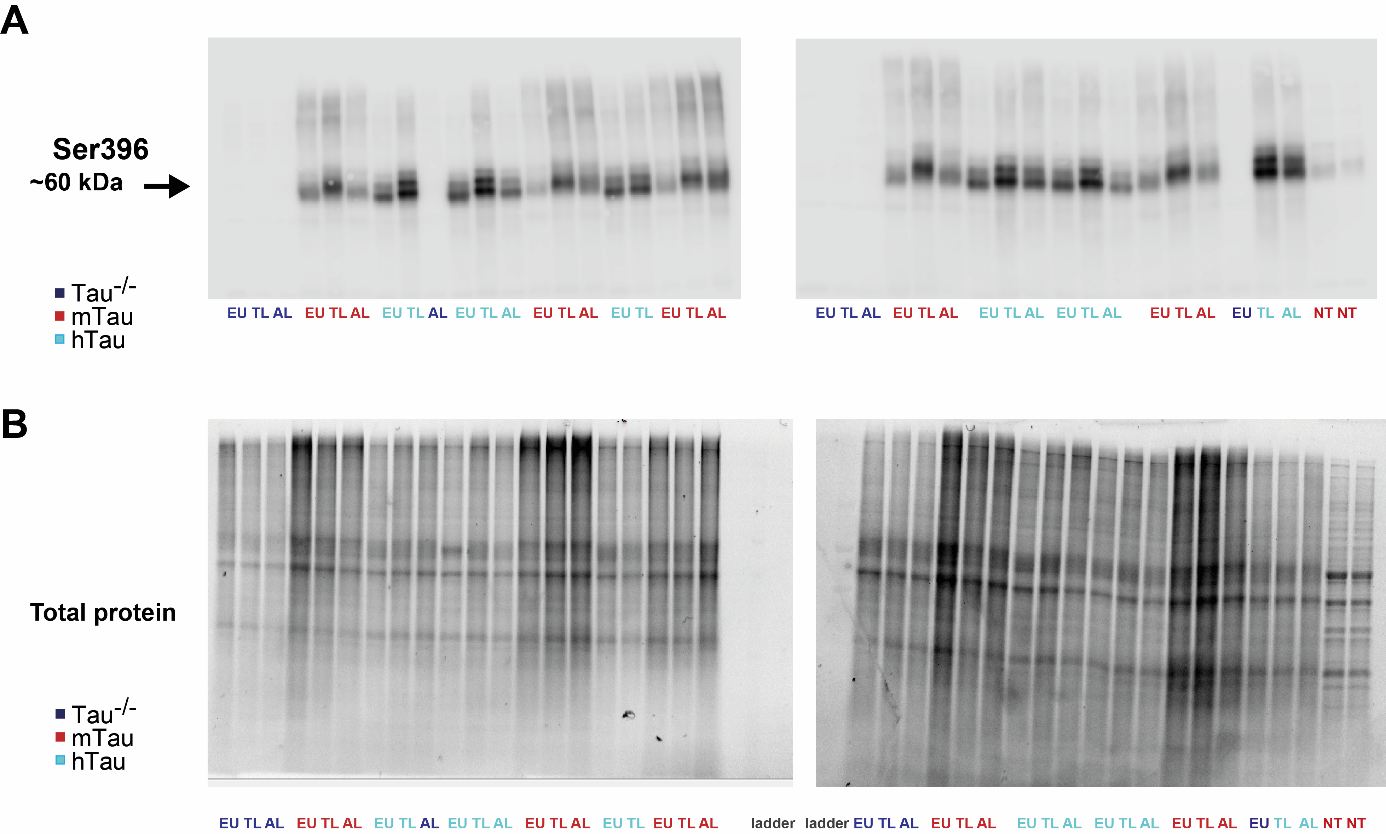
**

**Figure S5: Ser396 immunoblots and corresponding gels**

(**A**) Ser396 immunoblots for all Tau -/- (dark blue), mTau (red) and hTau (light blue) samples showed bands around 60kDa; indicated with arrow. (**B**) ECL protein staining of total protein levels was used for normalization.


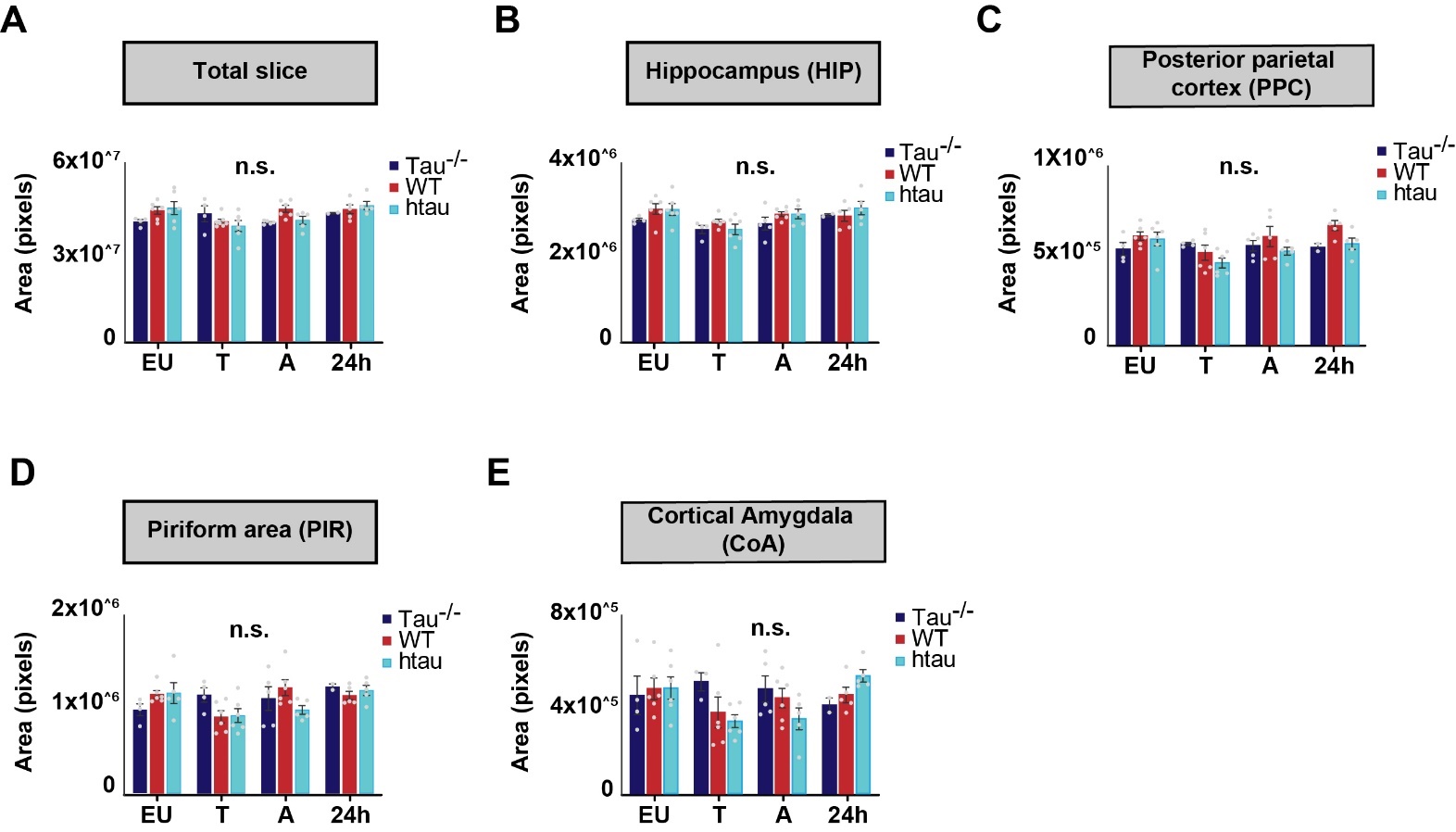


### Figure S6: Size of areas measured for total slice, PPC, HIP, PIR, CoA of EU, T, A, 24h Tau-/-, mTau and hTau mice

(**A-E**) The size of the areas measured for AT8 intensity did not differ between groups for the total slice, hippocampus, posterior parietal cortex, piriform cortex and cortical amygdala (2-way ANOVA F (6, 35) = 2,225, F (6, 51) = 0,9560, F (6, 35) = 1,494, F (6, 50) = 2,161, F (6, 36) = 1,798; p > 0.05).

###
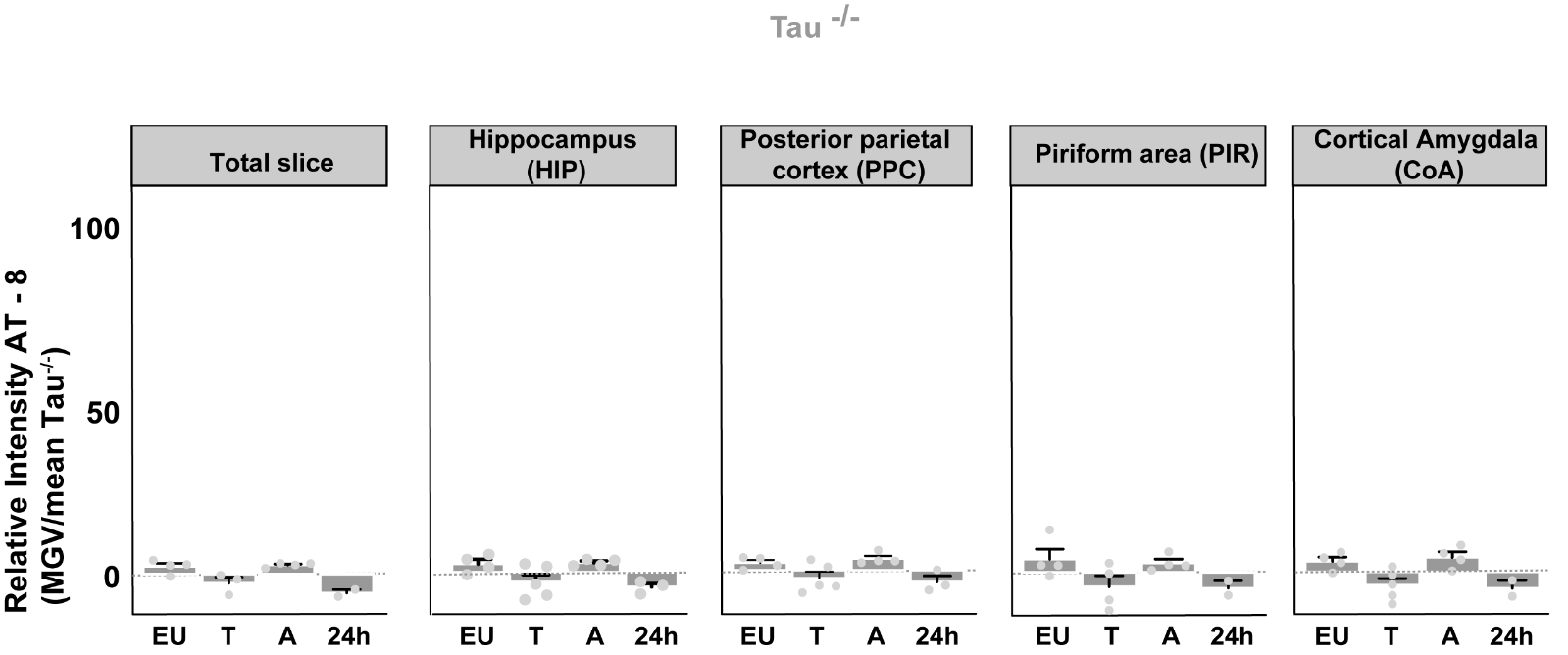


### Figure S7: AT8 intensity Tau-/- mice

Bar graphs of relative AT8 intensity (to mean Tau-/- MGV) of the total slice, Hippocampus (HIP), Posterior Parietal Cortex (PPC), Piriform Cortex (PIR) and Cortical Amygdala (CoA) of Tau^-/-^ Eutherm (EU), Torpor (T), Arousal (A) and 24h post arousal (24h) mice. Total slice: EU: n = 4, T: n = 3, A: n = 4, 24h n = 2; HIP: : EU: n = 4, T: n = 5, A: n = 4, 24h n = 3; PPC: EU: n = 4, T: n = 5, A: n = 4, 24h n = 3; PIR: EU: n = 4, T: n = 4, A: n = 4, 24h n = 2; CoA: EU: n = 4, T: n = 5, A: n = 4, 24h n = 2.


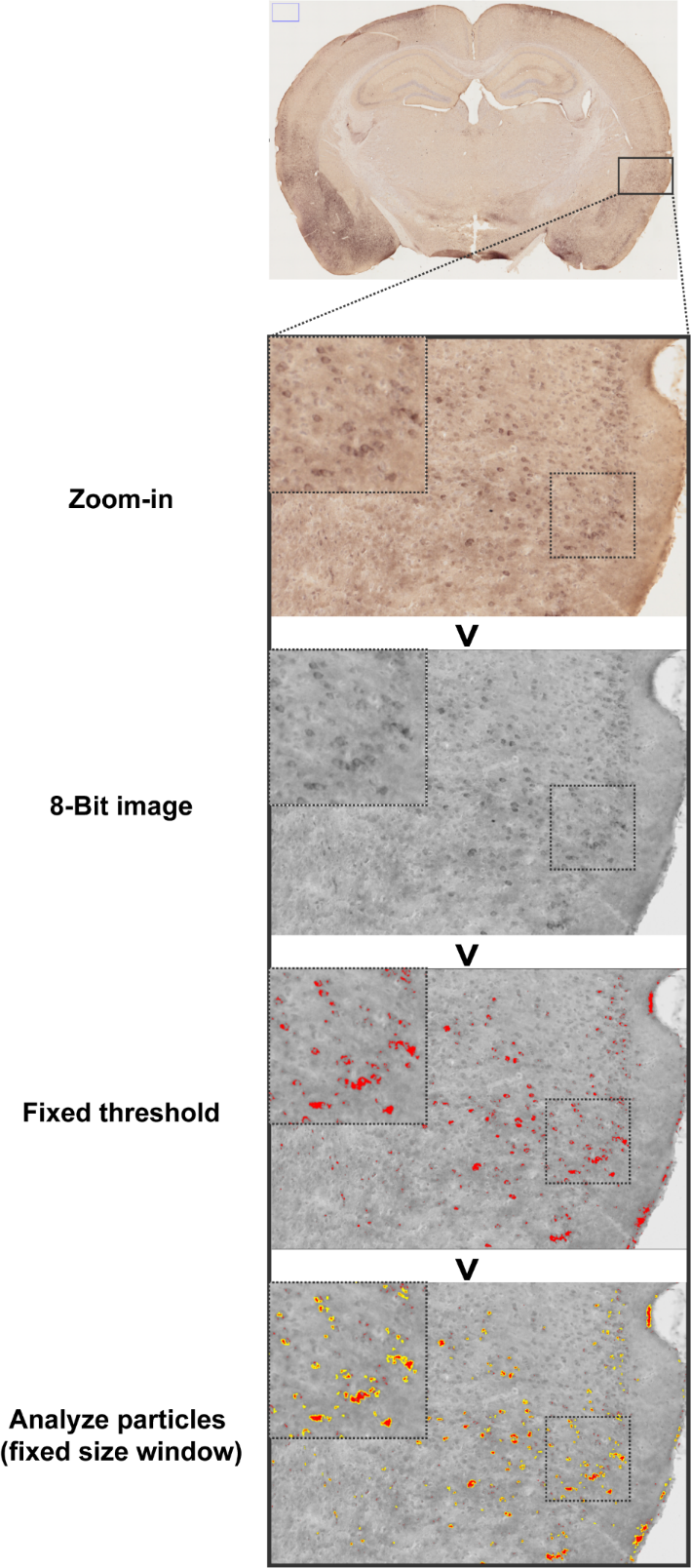


**Figure S8: measuring PT-like structures**

Pretangle-like structures (PT-like structures) with somato-dendritic accumulation of p-tau were measured for the total slice and per zoom in of HIP, PPC, PIR (illustrated in this example analyses) and CoA of EU, A and 24h mtau and htau mice, using Fiji. A fixed zoom in of 75% was made and the area of interest was converted to an 8-bit image. Then a fixed threshold (the same for all samples) was applied and using the analyze particles tool in Fiji, particles in a fixed size window (the same for all samples) were measured and counted as PT-like structures.


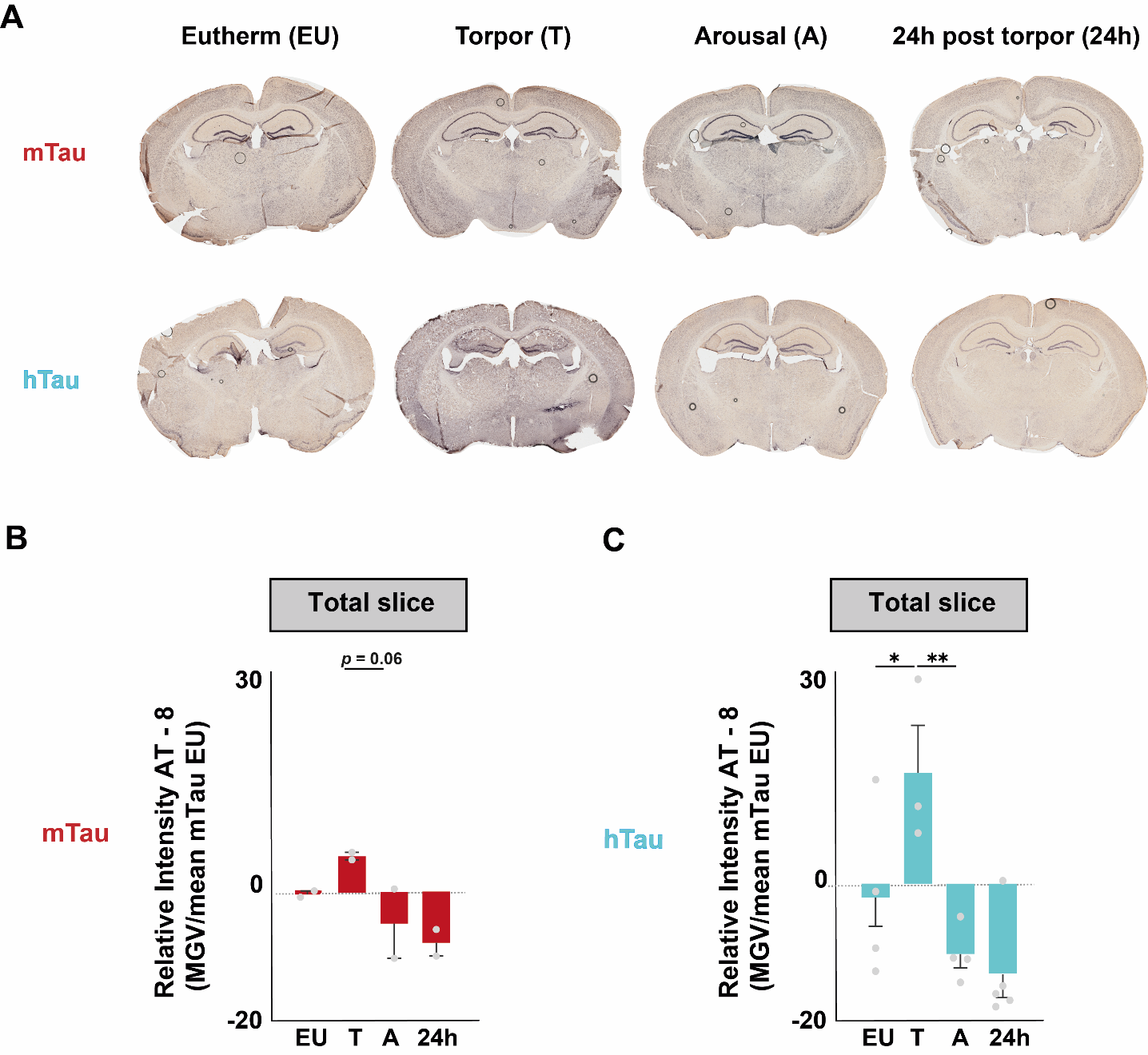
**Figure S9: Immunohistological analysis of phospho-tau (AT100) levels in mtau and htau brains**

### (A) Representative coronal slices of eutherm (EU), torpor (T), arousal (A), and 24 post-torpor (24h) mtau and htau mice, stained with AT100. (B, C) Quantification of AT100 total slice intensity (corrected for mean mtau EU mean gray value) shows an increase in tau phosphorylation during torpor for both mtau (B) and htau (C) (EU vs. TL; mtau; *p* = 0.239, t = 1.382, df = 4; htau: *p* = 0.025 t = 2.565, df = 12; 1-Way ANOVA, post-hoc Fisher’s LSD) which is reversed upon arousal (EU vs. A; mtau: *p* = 0.06, t = 2.576, df = 4; htau: *p* = 0.0029, t = 3.725, df = 12).
